# Characterising resuscitation promoting factor fluorescent-fusions in mycobacteria

**DOI:** 10.1186/s12866-018-1165-0

**Published:** 2018-04-12

**Authors:** Iria Uhía, Nitya Krishnan, Brian D. Robertson

**Affiliations:** MRC Centre for Molecular Bacteriology and Infection, Department of Medicine, Imperial College London, London, SW7 2AZ UK

**Keywords:** Rpfs, Mycobacteria, Tuberculosis, Fluorescent fusions, Microscopy

## Abstract

**Background:**

Resuscitation promoting factor proteins (Rpfs) are peptidoglycan glycosidases capable of resuscitating dormant mycobacteria, and have been found to play a role in the pathogenesis of tuberculosis. However, the specific roles and localisation of each of the 5 Rpfs in *Mycobacterium tuberculosis* remain mostly unknown. In this work our aim was to construct fluorescent fusions of *M. tuberculosis* Rpf proteins as tools to investigate their function.

**Results:**

We found that Rpf-fusions to the fluorescent protein mCherry are functional and able to promote cell growth under different conditions. However, fusions to Enhanced Green Fluorescent Protein (EGFP) were non-functional in the assays used and none were secreted into the extracellular medium, which suggests Rpfs may be secreted via the Sec pathway. No specific cellular localization was observed for either set of fusions using time-lapse video microscopy.

**Conclusions:**

We present the validation and testing of five *M. tuberculosis* Rpfs fused to mCherry, which are functional in resuscitation assays, but do not show any specific cellular localisation under the conditions tested. Our results suggest that Rpfs are likely to be secreted via the Sec pathway. We propose that such mCherry fusions will be useful tools for the further study of Rpf localisation, individual expression, and function.

**Electronic supplementary material:**

The online version of this article (10.1186/s12866-018-1165-0) contains supplementary material, which is available to authorized users.

## Background

Resuscitation promoting factor proteins (Rpfs) are peptidoglycan glycosidases able to resuscitate dormant bacteria. After the first description in *Micrococcus luteus* [1], five homologs were found in *Mycobacterium tuberculosis*, which can reactivate growth of dormant mycobacteria [2, 3]. They share a conserved Rpf domain, a 70 amino-acid region responsible for biological activity [4]. Knockouts of these proteins have been extensively studied [5–7]. The muralytic activity of these proteins [8, 9] may serve in peptidoglycan remodelling, although it is also proposed that the muropeptides produced modulate innate immune responses in the host, or activate cell resuscitation pathways [2, 10–12]. Despite the importance of these proteins in resuscitation of the important pathogen *M. tuberculosis*, little is known about their individual, specific roles, or localisation in the cell. Structural studies on RpfB, E and C catalytic domains shows a high degree of similarity, but also differences among these five proteins [13–16] . All Rpfs are expressed in early exponential and resuscitation phases, but are differentially expressed under stress [17], possibly due to differential regulation [18–22], suggesting their roles do not completely overlap.

Putative signal sequences at the amino terminus suggests they function extracellularly [23]. RpfB and RpfE interact with the cell wall hydrolase RipA, and RpfB and RipA colocalise at the septum of dividing cells, suggesting RpfB–RipA interactions are involved in separation of daughter cells during reactivation [12, 24–26].. The localisation and specific roles of the other Rpfs is not clear. Purified RpfE induces maturation of dendritic cells in mice, and it was suggested (but not demonstrated) that other Rpfs have this activity [27]. RpfA, RpfC and RpfE were found in culture filtrates of *M. tuberculosis* [28, 29], showing they are, at least in part, secreted into the extracellular medium, where they might exert autocrine and/or paracrine signalling functions. RpfC has also been found in membranes [29, 30] suggesting multiple locations. Recently, His-tagged *M. tuberculosis* RpfA, RpfB, RpfD and RpfE overproduced in *M. smegmatis* were detected in the culture supernatant by ELISA [31].

Fusion of Rpfs to fluorescent proteins would help localise them within the cell and give indications about function and possible distinct roles. To date only the localisation of RpfB fused to RFP has been communicated [24]. In the work reported here, we tested fusions of the five *M. tuberculosis* Rpfs to two different fluorescent proteins, EGFP and mCherry. We found that all fusions to mCherry, but none of the EGFP fusions, were functional. These results make Rpf-mCherry fusions interesting tools for studying resuscitation in mycobacteria.

## Results

### Fusion of Rpfs to fluorescent proteins and microscopic analysis

#### *EGFP* fusions

We amplified the five *rpf* genes from *M. tuberculosis* H37Rv and constructed C-terminal translational fusions to EGFP. This way, the N-terminus of each *rpf* gene was unmodified, maintaining putative signal sequences. We transformed *M. smegmatis* mc^2^155, a fast growing non-pathogenic mycobacteria used as a surrogate for *M. tuberculosis*, with plasmids harbouring each of the five constructs. The strains producing Rpf-EGFP fusions grew at the same rate as those expressing EGFP alone (Additional file 1: Figure S1a). Each Rpf-EGFP protein was expressed from a replicative plasmid (pSTetRO-*rpfA/B/C/D/E*-*egfp*) under control of a tetracycline inducible promoter.

**Fig. 1:**
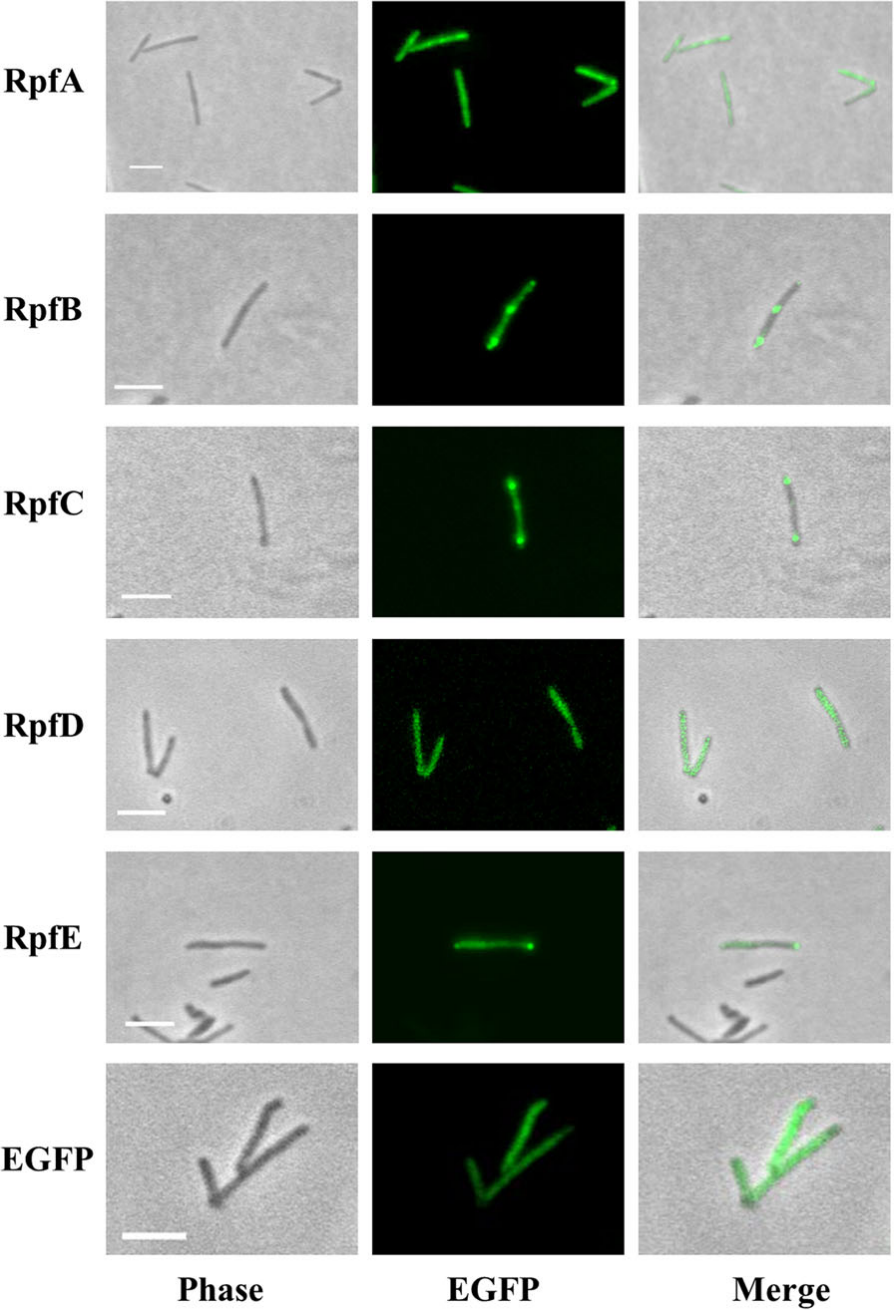
Rpf-EGFP localisation using static microscopy. The Rpf proteins fused to EGFP are widespread all along the cell body. In around 12% of cells, RpfB-EGFP, RpfC-EGFP and RpfE-EGFP accumulation is observed in the septum area and/or the tips of the cells; examples of this phenotype are shown. For RpfA-EGFP and RpfD-EGFP, the diffuse pattern was the only one observed. Scale bar: 5 μm. EGFP indicates a control producing EGFP alone

Using static microscopy the five fusion proteins appeared distributed along the cell body, but in a fraction of cells RpfB-EGFP, RpfC-EGFP and RpfE-EGFP localised at the tips and septum, the only areas of peptidoglycan production and growth in mycobacteria (Fig. 1). However, only around 12% of cells showed this localisation (12.8% for RpfB-EGFP (*n* = 219), 12.7% for RpfC-EGFP (*n* = 134) and 11.5% for RpfE-EGFP (*n* = 139)), raising the question whether this was due to over-production of the fusion proteins, although RpfB has been reported to localise to the septum [24].

We were unable to see any evidence of localisation of the proteins using time-lapse microscopy of cells growing in a microfluidic chamber; instead fluorescence was distributed along the cell body (Additional file 2: Movie S1). We could see transient accumulations of fluorescence at the septum in some cells, but in other cells, it became apparent that the accumulation of fluorescence at the tips or septum was most likely caused by precipitated proteins since these cells did not grow (Additional file 3: Movie S2).


Additional file 2:**Movie S1.** Localisation of RpfE-EGFP fusions in *M. smegmatis* (time lapse microscopy). RpfE-EGFP is shown as an example; all the Rpfs fused to EGFP present widespread localisation. (AVI 168 kb)



Additional file 3:**Movie S2.** Putative RpfB-EGFP inclusion bodies in *M. smegmatis* (time lapse microscopy). (AVI 150 kb)


All Rpfs have predicted signal peptides at the N-terminus [23], and are most probably secreted by the general secretion (Sec) pathway. Consequently, proteins must be completely folded in the periplasm or cell wall area [32]. However, EGFP is likely to be inefficiently folded in this location, due to the presence of cysteine groups able to form interchain disulphide bonds in this oxidizing environment, therefore blocking its maturation [33]. This in turn probably affects the localisation and activity of EGFP-fused Rpfs. Therefore, as an alternative, we fused the Rpfs to mCherry, a fluorescent protein that does not contain cysteine residues and hence should not be misfolded in the periplasm.

#### *mCherry* fusions

The *rpf* genes were fused to the N-terminus of *mcherry* and the fluorescent fusion proteins produced from replicative plasmids pMEND - *rpfA/B/C/D/E-mcherry*, under control of a tetracycline inducible promoter.

**Fig. 2:**
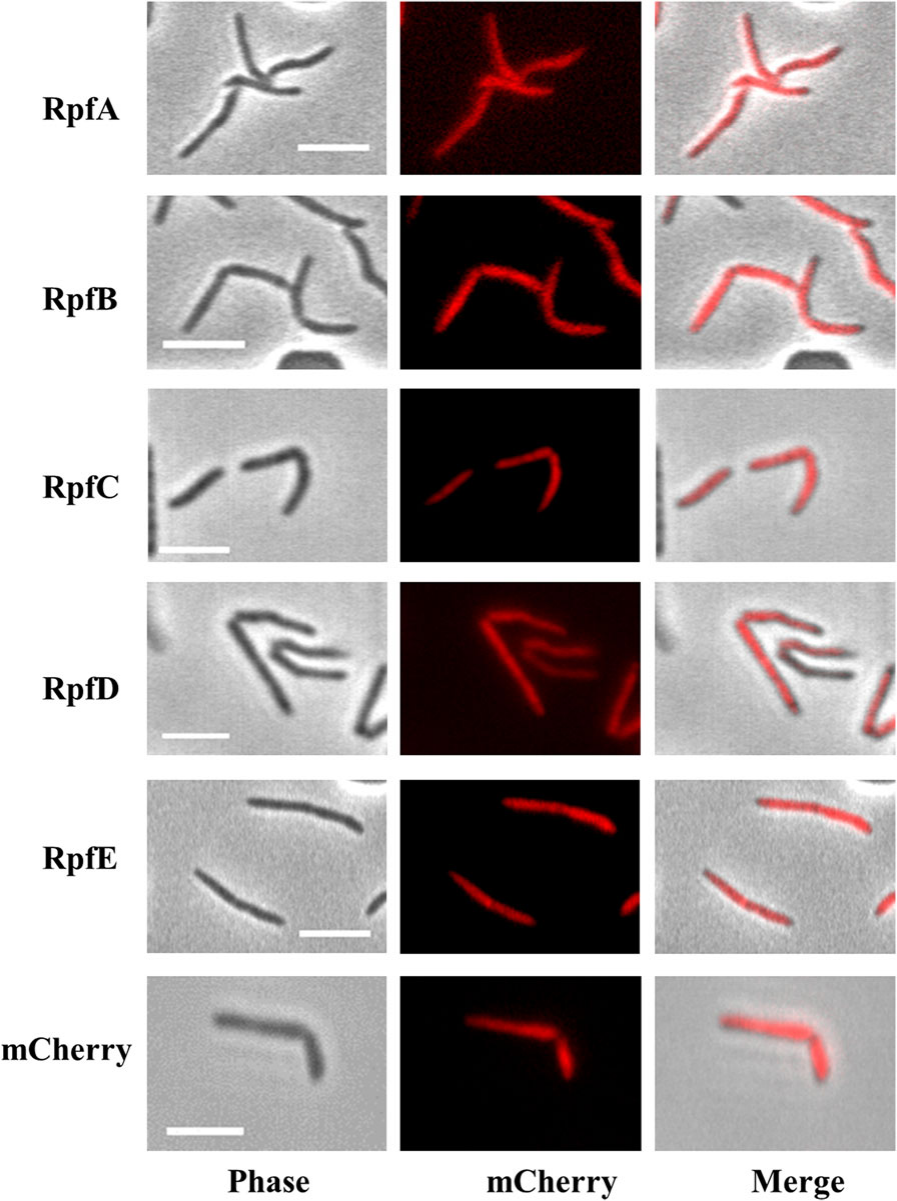
Rpf-mCherry localisation using static microscopy. Rpf proteins fused to mCherry are widespread all along the cell body. Scale bar: 5 μm. mCherry indicates a control producing mCherry alone


*M. smegmatis* transformed with each of the plasmids grows at the same rate as the control strain (Additional file 1: Figure S1b) and static microscopy did not show a specific localisation for any of the Rpfs; all of them diffused throughout the cell body (Fig. 2). In time lapse microscopy the results were very similar. As with EGFP fused proteins, we could sometimes see temporary accumulations of fluorescence in the septum area prior to division, especially for strains producing RpfA, RpfB and RpfE (Additional file 4: Movie S3).

Additional file 4:**Movie S3.** Localisation of RpfE-mCherry fusions in *M. smegmatis* (time lapse microscopy). RpfE-EGFP is shown as an example; all the Rpfs fused to mCherry present widespread localisation. (AVI 351 kb)


### Western blots of fluorescent fusion proteins

**Fig. 3:**
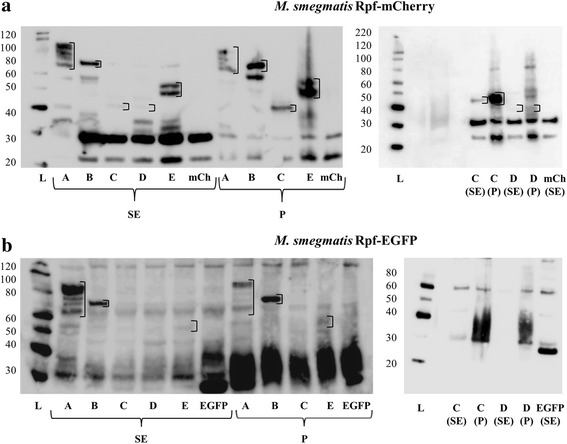
Localisation of Rpf-mCherry/EGFP fusions by western blot: protein extracts. The samples were extracted as detailed in Methods. SDS-PAGE gels were loaded with approximately 20 μg of soluble extract (SE; total proteins) and 5 μl of precipitated extract (P), previously resuspended in 50 μl of loading buffer. The letters A-E correspond to samples from RpfA-E-mCherry/EGFP-producing cultures, respectively. mCh and EGFP refer to the control strains harbouring control plasmids. The molecular weight ladder (L) values in kDa are shown on the left of each gel. Empty squares have been included encircling the bands of interest. **a** The left western blot performed with Rpfs-mCherry producing strains presents bands at the expected heights for all the fusion proteins in the SE and P. In some cases (RpfA and RpfE) more than one band appears per sample, most probably due to protein degradation/aggregation. The right blot contains repeat RfpC-mCherry samples and RpfD-mCherry pellet (that is not present on the left). The results show that these two proteins are present in both SE and P, but are more concentrated in the P fraction. **b** In the left blot performed with Rpfs-EGFP producing strains, RpfC-EGFP could not be identified in the SE or P. The right blot contains repeat RfpC-mCherry samples (as this protein could not be identified on the left) and RpfD-mCherry pellet (that was not present in the WB on the left). These two proteins could not be identified in the SE or P. Hypothetical molecular weight of the fusion proteins: RpfA-EFFP/mCherry (≈67 kDa), RpfB-EGFP/mCherry (≈65 kDa), RpfCEGFP/mCherry (≈45 kDa), RpfD-EGFP/mCherry (≈43 kDa), RpfE-EGFP/mCherry (≈46 kDa)

**Fig. 4:**
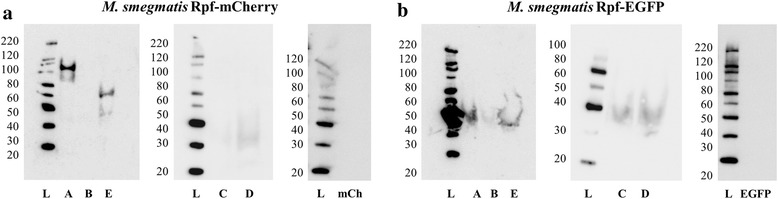
Localisation of Rpf-mCherry/EGFP fusions by western blot: culture supernatants. SDS-PAGE gels were loaded with 20 μl of concentrated culture supernatants (previously precipitated and resuspended in 40 μl of loading buffer; see Methods). The letters A-E correspond to samples from RpfA-E-mCherry/EGFP-producing cultures, respectively. mCh and EGFP refer to the control strains expressing fluorophore alone. The molecular weight ladder (L) values in kDa are shown on the left of each gel. **a** The western blots performed with culture supernatants of RpfA-mCherry and RpfE-mCherry producing strains present bands at the expected heights for the fusion proteins, as seen in soluble and precipitated extracts (Fig. 3). **b** In the western blots performed with culture supernatants of Rpf-EGFP producing strains, none of the samples present bands at the expected heights, indicating that probably none of the fusion proteins is being secreted to the extracellular medium. Hypothetical molecular weight of the fusion proteins: see Fig. 3 legend

Western blot was used to check the presence of fusion proteins in soluble extracts and precipitated fractions of disrupted cells, and in culture supernatants. Using an anti-mCherry antibody, we were able to detect proteins bands in soluble extracts and the precipitated fraction that matched the expected size for each fusion (Fig. 3a). Some of the proteins appear as multiple bands, probably due to protein degradation/aggregation. When we analysed the filtered culture supernatant, we were able to detect RpfA-mCherry and RpfE-mCherry proteins (Fig. 4a), confirming that these are secreted into the extracellular medium, as had previously been shown by proteomics and ELISA [28, 29, 31].

The detection of RpfA-mCherry and RpfE-mCherry in culture supernatants strongly suggests that these mCherry-fusions are efficiently translocated to their correct localisation in the cell. In contrast, when analysing Rpf-EGFP fusions, none were detected in the culture supernatant (Fig. 4b) and neither RpfC-EGFP nor RpfD-EGFP were detected in soluble and precipitated extracts (Fig. 3b), supporting our hypothesis that these fusions have impaired the function of the Rpf protein.

### Functionality of Rpf-mCherry fusions

**Fig. 5:**
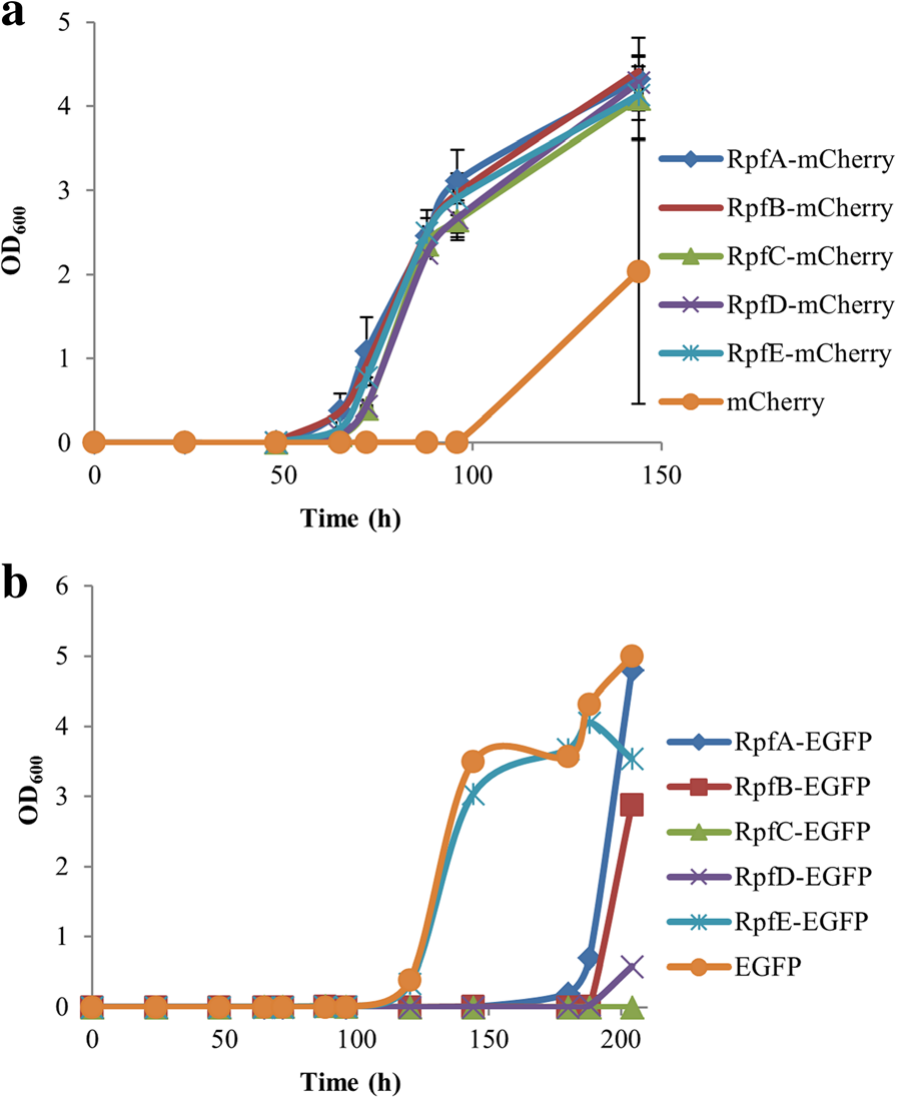
Functionality of Rpf-mCherry/EGFP fusions: nutritional shift-down assay. The assay was performed as detailed in Methods. **a** Rpf-mCherry fusions are functional: their overproduction stimulates the growth of stationary cells subjected to nutritional shift-down much faster than the overproduction of mCherry alone. Growth curves were made with data collected from 3 biological replicates. Error bars indicate standard error of the mean. **b** Rpf-EGFP fusions are not functional: their overproduction does not stimulate the growth of cells subjected to nutritional shift-down any better than the negative control overproducing EGFP. A representative growth curve (of at least 3 biological replicates) is shown for each strain

We tested the functionality of the fusions by checking the ability of each strain to resume growth after being subjected to nutritional shift-down, adapted from a published protocol [4]. Briefly, strains were grown in rich broth until stationary phase; then 100–600 cells per ml were subcultured in defined minimal broth. The lag phase of the cultures was substantially reduced when any of the five Rpfs-mCherry fusions was produced, in comparison to the strain producing mCherry alone (Fig. 5a), achieving OD values close to stationary phase (3) before the control starts to grow. There is some biological variability regarding the time when the mCherry strain begins to grow, ranging from 40 to 100 h after the Rpf-mCherry strains, but the results are highly reproducible. However, the mCherry strain does eventually reach the same OD, despite the longer lag phase and there is no statistical difference. Interestingly, the enhancement of growth was equal for all the overproduced Rpfs. A reduction in the lag phase was not observed for the EGFP-fused Rpfs (Fig. 5b). The growth curves shown are in this case a representative of at least 3 biological replicates for each strain and not mean values, as the different strains start to grow in a highly stochastic fashion in each experiment, with the control strain (EGFP) the first to grow. These results strongly suggest that Rpfs-EGFP fusions are not functional: growth inhibition due to overproduction of Rpfs-EGFP is unlikely; both sets of proteins (Rpfs-mCherry and Rpfs-EGFP) are under the control of the same promoter and induced under the same conditions, and western blot analysis shows the Rpfs-EGFP proteins are not produced in higher amounts.

### Functionality of Rpf-mCherry fusions under different types of stress

In addition to demonstrating that Rpf-mCherry fusions are functional in a nutritional shift-down assay, we also tested if their overproduction could resuscitate cells after they had been subjected to different stresses. We aimed to determine if specific Rpfs were required for particular stresses, or whether there are functional overlaps that might form a hierarchy.

Suboptimal growing conditions or the presence of antibiotics produce non-platable mycobacteria, which resume growth in a Rpf-dependent manner [21, 31, 34]. The different strains were subjected to acidic (pH 4.5) or osmotic stress conditions (1 M NaCl), or nutritional starvation (incubation in PBS), after which the capacity to stimulate the growth of non platable cells was calculated for each strain using the Most Probable Number (MPN) method (US Food and Drug Administration, 2010), and Colony Forming Units (CFU) counts.

As expected, cells under acidic, osmotic stress and nutrient starvation exhibit a less significant loss of viability (tested by CFUs) than those exposed to antibiotic stress [31], with a CFU loss of 2–6 fold for Phosphate Buffered Saline (PBS) and 1 M NaCl, and 3–13 fold for pH 4.5 after 6 days of incubation (data not shown). 6 days was chosen as the analysis point, since the number of platable cells remains stable for up to 7 additional days after this point, indicating that the cells are no longer dying due to the stress.

**Fig. 6:**
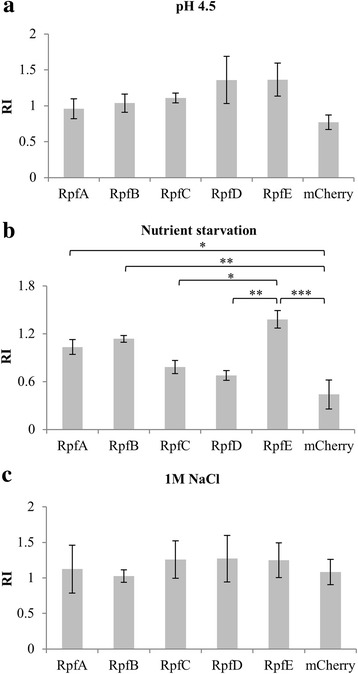
Induction of the growth of non platable cells by Rpf-mCherry producing strains after different stress conditions. The strains were subjected to three stress conditions and subsequently the potential for resuscitation of non platable cells was calculated for each strain as detailed in Methods and expressed as the Resuscitation Index (RI): log_10_(MPN)-log_10_(CFU). **a** Strains overproducing RpfD-mCherry and RpfE-mCherry show enhanced growth of non platable cells produced under pH 4.5, but this is not statistically significant. **b** The strain overproducing RpfE-mCherry enhances the growth of non platable cells produced under nutrient starvation to a greater extent, followed by RpfB-mCherry and RpfA-mCherry. **c** None of the strains overproducing Rpf-mCherry enhances the growth of non platable cells produced under 1 M NaCl. Three independent experiments were performed for each condition and strain. Error bars indicate standard error of the mean. Statistical differences in RI between strains were calculated using One Way Analysis of Variance followed by Bonferroni t-test. Cases in which differences were found to be statistically significant, are indicated by asterisks: ***P < 0.001; **P < 0.01; *P < 0.05

MPN and CFU values were analysed after stress. Cells previously exposed to acidic conditions (pH 4.5) show a tendency to increased capacity to resume growth of non platable cells when overproducing any of the Rpf-mCherry fusions compared to mCherry alone (Fig. 6a), with the highest numbers for the strains overproducing RpfE-mCherry and RpfD-mCherry (however the differences where not statistically significant by One Way Analysis of Variance under the conditions tested). This is in agreement with previous work that demonstrated that RpfE and RpfD are the two Rpfs with highest mRNA levels under acidic conditions [17]. In the case of nutrient starvation, RpfE-mCherry was again best at promoting growth of non platable cells, followed by RpfB and RpfA, the results being in this case statistically significant (Fig. 6b). For osmotic stress, there was no difference between the control strain and the overproducing strains (Fig. 6c).

## Discussion

The cellular localisation of four of the five Rpfs in *M. tuberculosis* remains unknown. As a prelude to investigating this, we have constructed and tested the functionality of two sets of Rpf fluorescent fusion proteins. Rpf-EGFP fusions gave a diffuse pattern of localisation when analysed using microscopy, with some of the proteins (RpfB-EGFP, RpfC-EGFP and RpfE-EGFP) showing a tendency to aggregate. None of the five EGFP- fusion proteins were functional, with none of them secreted into the extracellular medium; this suggests that Rpfs are secreted by the Sec pathway and therefore folded in the periplasmic area, where EGFP is known to be misfolded [33].

Rpf-mCherry fusions also did not show specific localisation in the cell, but in contrast to EGFP-fusions, none formed aggregates. Western blot showed all Rpf-mCherry proteins were found in the soluble and insoluble extracts of disrupted cells, with RpfA-mCherry and RpfE-mCherry also found in the culture supernatant. However, we were unable to detect any other Rpf proteins in the supernatant, contrary to published studies [28, 29, 31]. The explanation for this may be the different methodologies used. The fact that overproduced RpfA and RpfE were previously found to be the most abundant Rpfs in culture supernatants by Enzyme Linked Immunosorbent Assay (ELISA) [31], together with our results suggests that they may be the main Rpfs functioning extracellularly, whereas the rest may, at least transiently, attach to the cell envelope to exert their function. Producing Rpf-mCherry from their chromosomal loci with native promoters would give a more exact picture of the real situation in the cell. Tracking fluorescence production during infection or exposure to different stresses would also help better understand the role of these proteins, and expression from the native Rpf loci or from an integrative plasmid might help to obtain detailed localisation for each of these proteins. mCherry has potential for single-molecule localisation microscopy (SMLM) [35], but photoactivatable mCherry (PAmCherry) may be more suitable for photoactivated localisation microscopy (PALM) [36].

Rpf-mCherry proteins were all functional in terms of equal capacity to promote growth-recovery in a nutritional shift-down assay. Since all the Rpf-fusions were functional, we tested their capacity to promote growth of the non platable cells created under stress conditions. RpfE-mCherry and RpfD-mCherry seem to be most effective after pH 4.5 stress, and RpfE-mCherry after starvation stress; it has been suggested that this type of activity could be associated with tuberculosis reactivation [37]. The loss of platable cells (CFU) is low under these stress conditions, making interpretation difficult and differences between groups are not always statistically significant. We also used a parental strain producing native levels of Rpfs, and cannot rule out that they may interfere with testing of the contribution of individual overexpressed Rpf-mCherry fusions. The ideal would be to repeat these experiments with a parental strain deprived of the 5 Rpfs, but this is not currently available to us.

## Conclusions

In conclusion, the Rpf-mCherry fusions described here are a useful tool for the study of mycobacterial resuscitation factors, and their role in the biology of these organisms.

## Methods

### DNA manipulation, bacterial strains and growth conditions

The strains and the plasmids used in this work are listed in Additional file 5: Table S1. Primers used in this work are listed in Additional file 6: Table S2. *Mycobacterium smegmatis* mc^2^155 and derivatives were grown in defined liquid medium, either Middlebrook 7H9 (OADC supplemented, Difco), Sauton’s medium (0.05% potassium phosphate monobasic, 0.05% magnesium sulphate, 0.2% citric acid, 0.005% ferric ammonium citrate, 0.4% L-asparagine, 5% glycerol, 1‰ zinc sulphate solution; pH adjusted to 7) or Hartmans-de Bont minimal medium (Hartmans et al.*,* 2006), all supplemented with 0.025% tyloxapol (Sigma). Liquid cultures were grown aerobically at 37 °C in an orbital shaker at 180 rpm. For growth on solid medium mycobacteria were grown on Middlebrook 7H11 agar (OADC supplemented, Difco) incubated at 37 °C. When needed, hygromycin (50 μg/ml) and kanamycin (20 μg/ml) were used for plasmid selection and maintenance. Tetracycline (5 ng/ml) was used to induce mCherry (and fusion derivatives) production. *Escherichia coli* DH5α strain was used as a host for cloning. It was grown in LB medium at 37 °C in an orbital shaker at 180 rpm. LB agar plates were used. Hygromycin (150 μg/ml) and kanamycin (50 μg/ml) were used for plasmid selection and maintenance.

### Construction of plasmids

Plasmids harbouring each *rpf* gene fused to EGFP were constructed by amplifying each of the five *rpf* genes in *M. tuberculosis* H37Rv genome (*rpfA* (Rv0867c), *rpfB* (Rv1009), *rpfC* (Rv1884c), *rpfD* (Rv2389c) and *rpfE* (Rv2450c)) and cloning them in the EcoRI site of plasmid pST5552 [38]. In-frame fusions to EGFP were checked by sequencing. The promoter region of pST5552 was substituted by cloning the tetracycline-inducible promoter from plasmid pMEND [39] in the BamHI-EcoRI sites to generate plasmids pSTetRO-*rpfA/B/C/D/E*-*egfp*.

For the generation of plasmids with *rpfs* fused to mCherry protein, the five amplified genes were cloned into the BamHI–NdeI sites of pMEND-mCherry [40], which produced the fusion to the C-terminus of mCherry. A RBS was included in the 5′ primer upstream of each *rpf* start codon. The resulting plasmids pMEND-*rpfA/B/C/D/E-mcherry* have the fusion genes under the control of a tetracycline promoter.

Plasmids were electroporated into competent *M. smegmatis* mc^2^155 as described [41].

### Microscopy

Microscopy was performed in the Facility for Imaging by Light Microscopy (FILM) at Imperial College London. Time-lapse live cell microscopy was performed in B04A plates with an ONIX flow-control system (Merck-Millipore). Cells were loaded in the chamber at an OD_600_ of 0.1 from mid-exponential cultures in Hartmans-de Bont medium, and cultured at a continuous flow rate (1 psi) in a temperature-controlled chamber at 37 °C. Fluorescent fusion proteins were induced with tetracycline before loading the cells in the microfluidic chamber, where they continued to be perfused with the inducer. Images were captured every 15 min using a Zeiss Axiovert 200 inverted widefield microscope fitted with an EM-CCD (C9100–02) camera (Hammamatsu) controlled by HCImage software, using a 63X objective. Z-stacks were collected at 1 μm intervals to ensure in-focus images were collected. Images were analysed using Fiji image processing software [42].

For static microscopy, 20 μl samples of growing cultures were mounted on slides using Mowiol 4–88 (Calbiochem) previously to their visualization.

### Western blotting

For western blots of soluble extracts and precipitated fraction of disrupted cells, 10 ml cultures of the strains were grown in Hartmans-de Bont broth with the inducer tetracycline up to an OD_600_ of 1. The cultures were then centrifuged and resuspended in 500 μl PBS+ protease inhibitor (cOmplete, Sigma) and disrupted in a water bath sonicator. The soluble extract and the precipitated fraction were subsequently separated by centrifugation and protein concentration in the soluble extract was determined using the Pierce™ BCA Protein Assay Kit (Thermo Fisher). An equal amount of soluble proteins for all the samples was loaded in the gels for western blotting. The precipitated fractions were resuspended in the same volume for all the samples and a fixed volume was loaded on the gels. Western blots were performed using the NuPAGE Western blotting system (Thermo Fisher). SeeBlue Pre-stained Protein Standard (Thermo Fisher) was used as size standard. As a primary antibody, polyclonal rabbit anti-EGFP antibody (Thermo Fisher, dilution 1:1000) or polyclonal rabbit anti-mCherry antibody (Novus Biologicals, dilution 1:1000) were used, and as a secondary antibody we used goat anti-rabbit IgG-HRP (Santa Cruz Biotechnology, dilution 1:5000). The blots were developed using the SuperSignal West Femto Substrate Trial Kit (Thermo Fisher) in a LAS-3000 Fuji Imager.

For the western blots of filtrated culture supernatants, 100 ml of each strain were grown in Hartmans-de Bont broth without tween 80 and with the inducer tetracycline up to an OD_600_ of approximately 0.6. Protease inhibitor was added to the cultures 30 min before collecting, centrifuging and filtering the supernatants (0.22 μm). The proteins in the supernatants were concentrated by Ion-Exchange chromatography, adapting a published protocol [4]. The pI of the Rpfs-mCherry and Rpfs-EGFP fusion proteins was theoretically calculated using the Compute pI/Mw tool on the ExPASy Server [43]. It was found in all cases to be below the pH of the buffers (7.5) so the anion exchanger DEAE-Sepharose was used. 2 ml columns were washed with 5× volumes of water and then equilibrated with 5× volumes of buffer A (20 mM TrisHCl, pH 7.5; 20 mM KCl, 1 mM EDTA, 1 mM DTT). The supernatants were passed through the column and eluted with 3× volumes of buffer B (buffer A with 1 M NaCl). 2 ml samples of the first eluate were precipitated with 20% TCA and washed twice with acetone, dried and resuspended in loading buffer for its use in western blot, which was performed as previously explained.

The theoretical molecular weight of the fusion proteins was calculated using the Compute pI/Mw tool on the ExPASy Server [43].

### Nutritional shift-down assay

Strains were grown in 7H9 broth for 4 days in shaking, ensuring they reached stationary phase. They were then subcultured in 10 ml Hartmans-de Bont broth in the presence of the inducer tetracycline. The number of bacteria inoculated in Hartmans-de Bont was checked by CFUs counting and was determined to be between 100 and 600 cells/ ml in all cases. Cultures were grown in shaking and OD_600_ measured over time. Three biological replicates of each strain were analysed.

### Exposure to stress and calculation of viable counts

*M. smegmatis* strains harbouring each Rpf-mCherry or m-Cherry alone were grown in shaking up to mid-log phase (OD_600_ ≈ 0.6) in Sauton’s medium before being exposed to different stress conditions. For osmotic stress, a NaCl solution in water was added to the cultures at a 1 M final concentration. For acidic stress, cultures were washed in pH 4.5 Sauton and resuspended in the same medium. For nutrient starvation, cultures were washed twice in PBS-0.025% Tyloxapol (Sigma) and resuspended in the same solution. Samples for CFU counts were taken at this point and subsequently, the cultures were incubated for 6 days at 37 °C in static. After the incubation, the cultures were washed and resuspended in Sauton’s medium, and samples were taken for CFU and MPN counts and resuscitation index calculations.

Assessment of CFU counts was performed in serial dilutions of cultures in triplicate by using the standard droplet method [44] and subsequent incubation of the agar plates by 48 h.

For MPN counts, serial dilutions of 200 μl cultures samples were incubated in 96-well plates in Sauton’s medium containing the inducer tetracycline. Three wells were used for each dilution. The plates where incubated in static at 37 °C for 2 weeks and MPN calculated [45]. Three independent experiments were performed for each condition and strain. The potential for resuscitation of non platable cells of the different strains was expressed as the resuscitation index (RI): log_10_(MPN)-log_10_(CFU) [31]. Statistical differences in RI between strains were calculated using One Way Analysis of Variance (one-way ANOVA). We subsequently applied Bonferroni t-test, an all pairwise multiple comparisons procedure, to isolate the groups that differ from others. One-way ANOVA and Bonferroni t-test were applied using SigmaPlot (Systat Software, San Jose, CA).

## Additional files

Additional file 1: **Figure S1.** Growth of *M. smegmatis* Rpf-EGFP and Rpf-mCherry producing strains in 7H9. (**a**) Growth curve of strains overproducing Rpf-EGFP proteins and a control strain overproducing EGFP. (**b**) Growth curve of strains overproducing Rpf-mCherry proteins and a control strain overproducing mCherry. Growth curves were made with data collected from 3 biological replicates. Error bars indicate standard deviation. (PDF 16 kb)

Additional file 5: **Table S1.** Bacterial strains and plasmids used in this study. (PDF 27 kb)

Additional file 6: **Table S2.** Primers used in this study. (PDF 14 kb)

## Abbreviations

CFU: Colony Forming Units
EGFP: Enhanced Green Fluorescent Protein
ELISA: Enzyme Linked Immunosorbent Assay
MPN: Most Probable Number
PALM: Photoactivated localisation microscopy
PBS: Phosphate Buffered Saline
Rpfs: Resuscitation promoting factor proteins
SMLM: Single-molecule localisation microscopy
