# Supplementary material for: Characterising resuscitation promoting factor fluorescent-fusions in mycobacteria
Source: BMC Microbiol. 2018 Apr 12;18:30. doi: 10.1186/s12866-018-1165-0 (PMC5898023; doi:10.1186/s12866-018-1165-0)
Supplement: Supplementary file 4 — Figure S1. Growth of M. smegmatis Rpf-EGFP and Rpf-mCherry producing strains in 7H9. (a) Growth curve of strains overproducing Rpf-EGFP proteins and a control strain overproducing EGFP. (b) Growth curve of strains overproducing Rpf-mCherry proteins and a control strain overproducing mCherry. Growth curves were made with data collected from 3 biological replicates. Error bars indicate standard deviation. (PDF 16 kb) [file 12866_2018_1165_MOESM1_ESM.pdf]

a

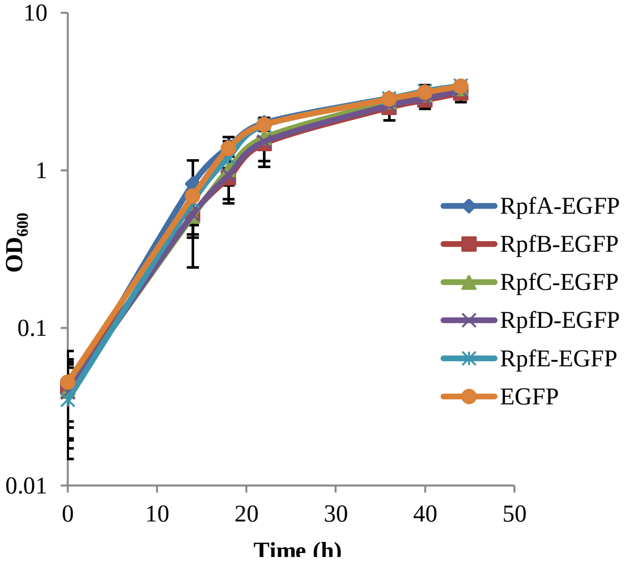

b

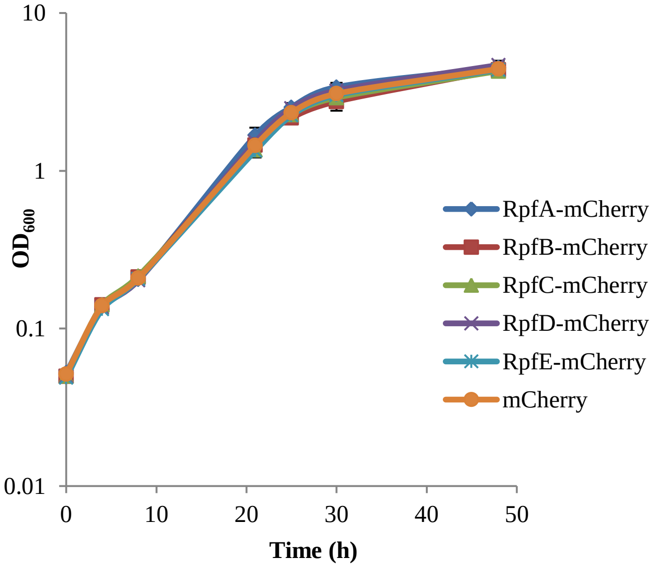

Additional figure 1. Growth of *M. smegmatis* Rpf-EGFP and Rpf-mCherry producing strains in 7H9. (**a**) Growth curve of strains overproducing Rpf-EGFP proteins and a control strain overproducing EGFP. (**b**) Growth curve of strains overproducing Rpf-mCherry proteins and a control strain overproducing mCherry. Growth curves were made with data collected from 3 biological replicates. Error bars indicate standard deviation.
