# Supplementary material for: Characterising resuscitation promoting factor fluorescent-fusions in mycobacteria
Source: BMC Microbiol. 2018 Apr 12;18:30. doi: 10.1186/s12866-018-1165-0 (PMC5898023; doi:10.1186/s12866-018-1165-0)
Supplement: Supplementary file 5 — Table S1. Bacterial strains and plasmids used in this study. (PDF 27 kb) [file 12866_2018_1165_MOESM5_ESM.pdf]

Additional Table 1. Bacterial strains and plasmids used in this study.

Strain/Plasmid	Description	Source
Escherichia coli DH5α	*F-, endA1, hsdR17 (rk-mk+), supE44, thi-1, recA1, gyrA, relA1, Δ(argF-lac)U169, deoRΦ80dlac, Δ (lacZ)M15*	[1]
M. smegmatis mc^{2}155	*ept-1,* mc^{2}6 mutant efficient for electroporation	[2]
pMEND-mCherry	Km^R, Hyg^R mycobacterial replicative expression vector pMEND containing *mcherry* under the control of a tetracycline inducible promoter.	[3]
pMEND-*rpfA-mcherry*	pMEND-mCherry containing *rpfA* gene from *M. tuberculosis* H37Rv fused to the N-terminus of *mcherry*.	This work
pMEND-*rpfB-mcherry*	pMEND-mCherry containing *rpfB* gene from *M. tuberculosis* H37Rv fused to the N-terminus of *mcherry*.	This work
pMEND-*rpfC-mcherry*	pMEND-mCherry containing *rpfC* gene from *M. tuberculosis* H37Rv fused to the N-terminus of *mcherry*.	This work
pMEND-*rpfD-mcherry*	pMEND-mCherry containing *rpfD* gene from *M. tuberculosis* H37Rv fused to the N-terminus of *mcherry*.	This work
pMEND-*rpfE-mcherry*	pMEND-mCherry containing *rpfE* gene from *M. tuberculosis* H37Rv fused to the N-terminus of *mcherry*.	This work
pST5552	Km^R mycobacterial replicative expression vector. Contains *egfp* under the control of a riboswitch-based theophilline inducible system.	[4]
pSTetRO-*egfp*	pST5552 containing *TetRO* promoter region from pMEND in the BamHI-EcoRI sites	This work
pSTetRO-*rpfA-egfp*	pSTetRO-*egfp* containing *rpfA* gene from *M. tuberculosis* H37Rv fused to the N-terminus of *efgp*	This work
pSTetRO-*rpfB-egfp*	pSTetRO-*egfp* containing *rpfB* gene from *M. tuberculosis* H37Rv fused to the N-terminus of *egfp*	This work
pSTetRO-*rpfC-egfp*	pSTetRO-*egfp* containing *rpfC* gene from *M. tuberculosis* H37Rv fused to the N-terminus of *egfp*	This work
pSTetRO-*rpfD-egfp*	pSTetRO-*egfp* containing *rpfD* gene from *M. tuberculosis* H37Rv fused to the N-terminus of *egfp*	This work
pSTetRO-*rpfE-egfp*	pSTetRO-*egfp* containing *rpfE* gene from *M. tuberculosis* H37Rv fused to the N-terminus of *egfp*	This work

Abbreviations: EGFP, enhanced green fluorescent protein; Hyg, hygromycin; Km, kanamycin.

1. Hanahan D. Studies on transformation of *Escherichia coli* with plasmids. J Mol Biol. 1983;166:557–80.

2. Snapper SB, Melton RE, Mustafa S, Kieser T, Jr WRJ. Isolation and characterization of efficient plasmid transformation mutants of *Mycobacterium smegmatis*. Mol Microbiol. 1990;4:1911–9.

3. Joyce G, Williams KJ, Robb M, Noens E, Tizzano B, Shahrezaei V, et al. Cell division site placement and asymmetric growth in mycobacteria. PLoS One. 2012;7:e44582.

4. Seeliger JC, Topp S, Sogi KM, Previti ML, Gallivan JP, Bertozzi CR. A riboswitch-based inducible gene expression system for mycobacteria. PLoS One. 2012;7:e29266.
