# Supplementary material for: Characterising resuscitation promoting factor fluorescent-fusions in mycobacteria
Source: BMC Microbiol. 2018 Apr 12;18:30. doi: 10.1186/s12866-018-1165-0 (PMC5898023; doi:10.1186/s12866-018-1165-0)
Supplement: Supplementary file 6 — Table S2. Primers used in this study. (PDF 14 kb) [file 12866_2018_1165_MOESM6_ESM.pdf]

Additional Table 2. Primers used in this study

Primer	Sequence (5'-3')	Use
mCherry R	cctcgatctcgaactcgtgg	Sequencing inserts in pMEND-mCherry. Reverse primer
pMIND MCS F	gctctcggtcaagcacgtcg	Sequencing inserts in pMEND-mCherry. Forward primer
pST R	cgtaggtcagggtggtcacg	Sequencing inserts cloned in EcoR I site of pSTetRO-*egfp*. Reverse primer
RpfA F cherry	ctagggatccaggaggtgatgagcatgagtggacgccaccgtaag	Amplyfying and cloning *rpfA gene* from *M. tuberculosis* into pMEND-mCherry (fusion to the N-terminus of *mcherry*)
RpfA R cherry	ctagcatatggccgatgacgtacggctgtg	As above
RpfB F cherry	ctagggatccaggaggtgatgagcatgttgcgcctggtagtcgg	Amplyfying and cloning *rpfB gene* from *M. tuberculosis* into pMEND-mCherry (fusion to the N-terminus of *mcherry*)
RpfB R cherry	ctagcatatggcgcgcacccgctcgtgcag	As above
RpfC F cherry	ctagggatccaggaggtgatgagcgtgcatcctttgccggccgac	Amplyfying and cloning *rpfC gene* from *M. tuberculosis* into pMEND-mCherry (fusion to the N-terminus of *mcherry*)
RpfC R cherry	ctagcatatggcgcggaatacttgcctgaatg	As above
RpfD F cherry	ctagggatccaggaggtgatgagcatgacaccgggtttgcttactac	Amplyfying and cloning *rpfD gene* from *M. tuberculosis* into pMEND-mCherry (fusion to the N-terminus of *mcherry*)
RpfD R cherry	ctagcatatgatcgtccctgctccccgaac	As above
RpfE F cherry	ctagggatccaggaggtgatgagcatgcccgtgggttggctttgg	Amplyfying and cloning *rpfE gene* from *M. tuberculosis* into pMEND-mCherry (fusion to the N-terminus of *mcherry*)
RpfE R cherry	ctagcatatggccgcggcggccgcagac	As above
RpfA F egfp	ctaggaattcatgagtggacgccaccgtaag	Amplifying and cloning *rpfA* gene from *M. tuberculosis* into pSTetRO-*egfp* (fusion to the N-terminus of *egfp*)
RpfA R egfp	ctaggaattcgccgatgacgtacggctgtg	As above
RpfB F egfp	ctaggaattcatgttgcgcctggtagtcgg	Amplifying and cloning *rpfB* gene from *M. tuberculosis* into pSTetRO-*egfp* (fusion to the N-terminus of *egfp*)
RpfB R egfp	ctaggaattcgcgcgcacccgctcgtgcag	As above
RpfC F egfp	ctaggaattcgtgcatcctttgccggccgac	Amplifying and cloning *rpfC* gene from *M. tuberculosis* into pSTetRO-*egfp* (fusion to the N-terminus of *egfp*)
RpfC R egfp	ctaggaattcgcgcggaatacttgcctgaatg	As above
RpfD F egfp	ctaggaattcatgacaccgggtttgcttactactgc	Amplifying and cloning *rpfD* gene from *M. tuberculosis* into pSTetRO-*egfp* (fusion to the N-terminus of *egfp*)
RpfD R egfp	ctaggaattcatcgtccctgctccccgaac	As above
RpfE F egfp	ctaggaattcatgcccgtgggttggctttg	Amplifying and cloning *rpfE* gene from *M. tuberculosis* into pSTetRO-*egfp* (fusion to the N-terminus of *egfp*)
RpfE R egfp	ctaggaattcgccgcggcggccgcagac	As above
TetRO F	ctagggatcctaagtcattaaagttgcactttatcatcgataactttatc	Amplifying TetRO promoter region from pMEND and cloning in BamHI-EcoRI sites of pST5552
TetRO R	ctaggaattcacctccttaggtcagtcaggattccacgatgag	As above

Restriction sites are underlined.
